# The recommendations of a consensus panel for the screening, diagnosis, and treatment of neurogenic orthostatic hypotension and associated supine hypertension

**DOI:** 10.1007/s00415-016-8375-x

**Published:** 2017-01-03

**Authors:** Christopher H. Gibbons, Peter Schmidt, Italo Biaggioni, Camille Frazier-Mills, Roy Freeman, Stuart Isaacson, Beverly Karabin, Louis Kuritzky, Mark Lew, Phillip Low, Ali Mehdirad, Satish R. Raj, Steven Vernino, Horacio Kaufmann

**Affiliations:** 1 Harvard Medical School and Beth Israel Deaconess Medical Center, Boston, MA USA; 2 National Parkinson Foundation, Miami, FL USA; 3 Vanderbilt University Medical Center, Nashville, TN USA; 4 Duke University Hospital and Central Carolina Hospital, Durham, NC USA; 5 Parkinson’s Disease and Movement Disorders Center of Boca Raton, Boca Raton, FL USA; 6 University of Toledo Medical Center, Toledo, OH USA; 7 University of Florida College of Medicine, Gainesville, FL USA; 8 Keck/USC School of Medicine, Los Angeles, CA USA; 9 Mayo Clinic, Rochester, MN USA; 10 Saint Louis University Hospital, St. Louis, MO USA; 11 University of Calgary, Calgary, BC Canada; 12 University of Texas Southwestern Medical Center, Dallas, TX USA; 13 New York University Langone Medical Center, New York, NY USA

**Keywords:** Neurogenic orthostatic hypotension, Supine hypertension, Autonomic dysfunction, Droxidopa, Midodrine, Fludrocortisone

## Abstract

Neurogenic orthostatic hypotension (nOH) is common in patients with neurodegenerative disorders such as Parkinson’s disease, multiple system atrophy, pure autonomic failure, dementia with Lewy bodies, and peripheral neuropathies including amyloid or diabetic neuropathy. Due to the frequency of nOH in the aging population, clinicians need to be well informed about its diagnosis and management. To date, studies of nOH have used different outcome measures and various methods of diagnosis, thereby preventing the generation of evidence-based guidelines to direct clinicians towards ‘best practices’ when treating patients with nOH and associated supine hypertension. To address these issues, the American Autonomic Society and the National Parkinson Foundation initiated a project to develop a statement of recommendations beginning with a consensus panel meeting in Boston on November 7, 2015, with continued communications and contributions to the recommendations through October of 2016. This paper summarizes the panel members’ discussions held during the initial meeting along with continued deliberations among the panel members and provides essential recommendations based upon best available evidence as well as expert opinion for the (1) screening, (2) diagnosis, (3) treatment of nOH, and (4) diagnosis and treatment of associated supine hypertension.

**Electronic supplementary material:**

The online version of this article (doi:10.1007/s00415-016-8375-x) contains supplementary material, which is available to authorized users.

## Introduction

Neurogenic orthostatic hypotension (nOH) is a prevalent disorder [1, 2]. Clinicians from a variety of specialties need to be well informed about diagnosis and management of nOH as it carries a significant burden of morbidity and has also been associated with increased mortality [3–5]. Clinicians should, therefore, familiarize themselves with this condition and evolving management options, which offer substantial symptomatic improvement. This communication will focus primarily on nOH, while recognizing that orthostatic symptoms are often multifactorial: i.e., patients with nOH may have their symptoms worsened by medications, hypovolemia, intrinsic cardiovascular disease, and other factors [6, 7].

The literature on nOH is fraught with inconsistencies in the definition and methods of diagnosis, and suffers from a lack of evidence-based guidelines to direct clinicians towards ‘best practice’. As nOH is a subset of orthostatic hypotension (OH), it should be noted that patients with nOH and OH may experience the same symptoms but for different reasons. The following consensus definition of OH was devised by the American Autonomic Society and the American Academy of Neurology, and endorsed by the European Federation of Autonomic Societies and the World Federation of Neurology: OH is “…a sustained reduction of systolic blood pressure of at least 20 mmHg or diastolic blood pressure of 10 mmHg, or both, within 3 min of standing or head-up tilt to at least 60° on a tilt table” [8]. In addition to meeting the definition for OH, patients with nOH have impairment of the autonomic nervous system that is characterized by failure to provide adequate autonomic postural responses, most prominently systemic vasoconstriction and a compensatory increase in heart rate sufficient to maintain blood pressure. This deficit is, in large part, attributed to insufficient norepinephrine release from sympathetic nerves [9]. In addition, many patients with nOH also suffer from supine hypertension, which further confounds therapy because pharmacologic treatments to normalize standing blood pressure may worsen supine hypertension [10]. Differences in study designs and endpoints, as well as a paucity of data, preclude achieving definitive evidence-based treatment regimens through a systematic review of trials [11–22]. To move forward in an effective and safe manner, consensus is required on the basics of screening, diagnosing, and treating patients with nOH. Thus, an expert opinion statement to these basic approaches is needed.

To address this issue, the American Autonomic Society and the National Parkinson Foundation jointly held an initial consensus panel meeting to formulate essential recommendations into a working guideline for the screening, diagnosis, and treatment of nOH and associated supine hypertension. Results of the discussions held during the meeting, along with continued deliberations among the panel participants are presented here along with recommendations in each of the topic areas. Whereas most patients with disorders typically associated with nOH are seen by cardiologists or neurologists, particularly movement disorder specialists, the first point of contact and potential for identification of nOH is with the primary care clinicians. Hence, the following consensus commentary is meant to provide guidance for all clinicians who might encounter and/or ultimately manage nOH.

## Screening for nOH

There is no standardized or recommended screening protocol for patients that present with symptoms of orthostatic hypotension. Presenting symptoms and signs of OH include postural lightheadedness or dizziness, the sensation of blacking out, and falls with or without syncope. Less common symptoms include orthostatic cognitive dysfunction (executive function worsens significantly during the orthostatic challenge in patients with autonomic dysfunction, possibly because of transient frontal lobe hypoperfusion), mental dulling, generalized weakness, neck pain or discomfort in the suboccipital and paracervical region (‘coat hanger’ configuration—a manifestation of hypotension-induced ischemia of the strap muscles of the neck), or platypnea (dyspnea while standing due to OH causing inadequate perfusion of ventilated lung apices or ventilation perfusion mismatch) [23–27]. Thus, screening for nOH starts with questions to identify the symptoms of OH followed by measurement of blood pressures from the supine (or seated) to standing position (recommended specific technique for timing of blood pressure measurements is detailed below) [6]. It may be beneficial if screening questionnaires and blood pressure measurements are conducted by a qualified non-physician (such as a nurse) prior to the patient’s visit with the clinician. Patients in the following five categories need to be routinely screened for OH:

1. Patients suspected of, or diagnosed with any neurodegenerative disorder associated with autonomic dysfunction, including Parkinson’s Disease (PD), Multiple System Atrophy (MSA), Pure Autonomic Failure (PAF), or Dementia with Lewy Bodies (DLB);
2. Patients who have reported an unexplained fall or have had an episode of syncope;
3. Patients with peripheral neuropathies known to be associated with autonomic dysfunction (e.g., diabetes, amyloidosis, HIV);
4. Patients who are elderly (≥70 years of age) [28] and frail or on multiple medications;
5. Patients with postural (orthostatic) dizziness or non-specific symptoms that only occur when standing.

Patients in each of these groups have a higher risk of OH/nOH when compared to the normal population [29].

For these five categories of patients, clinicians should ask about cardinal symptoms of OH, their frequency and severity, how long they can stand, and the effect of symptoms on their activities of daily living. Questions about symptoms should also note the time of day when the symptoms occur, as symptoms of OH/nOH are most likely to occur in the morning and after meals [30–32]. A more complete list of questions to use when screening patients for OH/nOH is presented in Table 1. If a patient gives a positive response to one or more of the questions listed in Table 1, they should be considered as being at risk for OH/nOH and a more complete evaluation including orthostatic vital signs needs to be conducted to confirm a diagnosis of OH/nOH. From a practical standpoint, at the minimum, the patient should be asked a variation of question 10 “Do you have symptoms when you stand up or within 3–5 min of standing and get better when you sit or lay down?” Because falls are of such consequence to aging patients, a specific question about circumstances of falls is appropriate. Clinicians must recognize that some patients are reluctant to admit symptoms of OH or falls, fearing that they may lose their autonomy due to family member insistence on enhancing their safety.

**Table 1:** Screening questions for suspected OH/nOH

Question	Screening questions*
1	Have you fainted/blacked out recently?
2	Do you feel dizzy or lightheaded upon standing?
3	Do you have vision disturbances when standing?
4	Do you have difficulty breathing when standing?
5	Do you have leg buckling or leg weakness when standing?
6	Do you ever experience neck pain or aching when standing?
7	Do the above symptoms improve or disappear when you sit or lay down?
8	Are the above symptoms worse in the morning or after meals?
9	Have you experienced a fall recently?
10	Are there any other symptoms you commonly experience when you stand up or within 3–5 min of standing and get better when you sit or lay down?

* Any positive response should prompt further investigation with orthostatic blood pressure measurements

## Diagnosis of nOH in individuals who screen positive for OH

**Fig. 1:**
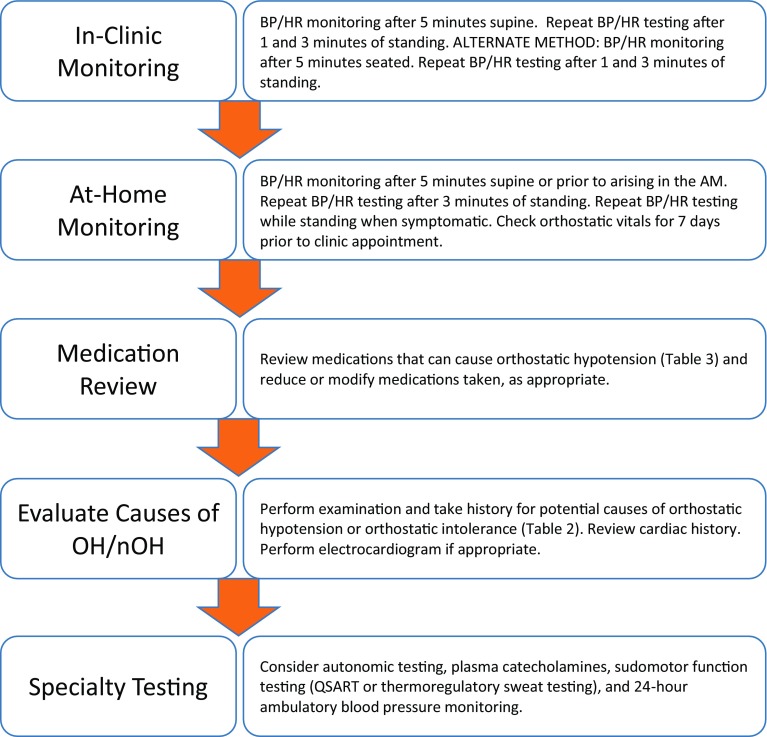
Stepwise approach to the diagnosis of nOH

After screening to identify a patient as being at risk for OH, an accurate assessment of the underlying cause(s) is required to determine the appropriate treatment recommendation. A stepwise approach is recommended for diagnosis of nOH, specifically starting with measurement of orthostatic blood pressure and heart rate, followed by more detailed autonomic testing in select cases (Fig. 1).

### Diagnostic tests for nOH

#### Blood pressure testing

The hallmark test for OH is measurement of change in blood pressure from supine, after at least 5 min of rest, to standing (or head-up tilt [HUT]) [33]. Current guidelines define OH as a sustained fall of systolic blood pressure of at least 20 mmHg or a diastolic blood pressure of 10 mmHg within 3 min of standing (or HUT) [8]. However, in patients with supine hypertension (a supine systolic blood pressure ≥150 mmHg or diastolic blood pressure ≥90 mmHg), a 30 mmHg decrease in systolic blood pressure or 15-point fall in diastolic blood pressure may be a more appropriate criterion for patients with nOH as the magnitude of blood pressure fall is dependent on the baseline blood pressure [8].

#### A practical stepwise approach to orthostatic blood pressure and heart rate testing

##### In-clinic monitoring of blood pressure and heart rate

The recommended gold standard measurement of OH includes having patients rest in the supine position for at least 5 min and then stand for 3 min, with blood pressure measurements taken just prior to standing and at both 1 and 3 min of standing [33]. However, in facilities in which this method is impractical, a seated-to-standing blood pressure can be performed as an acceptable alternative. In the alternative method, patients sit for at least 5 min and then stand for 3 min, with blood pressure measured just prior to standing and at 1 and 3 min of standing. If the test is positive (i.e., ≥20/10 mmHg decline in blood pressure with standing), the subject has OH. However, if the test is negative but symptoms are strongly suggestive of OH, a supine-to-standing blood pressure test (or HUT) should be considered. As some individuals with OH are at risk for falls when standing suddenly, testing staff should be aware of this possibility and take appropriate precautions.

Even this appropriate testing of blood pressure will not identify all patients with OH, since variability in meals, hydration, time of day, and medications can affect the orthostatic change in blood pressure. Additionally, there is a subgroup of patients who manifest symptoms of OH beyond the 3-min interval, defined as delayed OH [34], which may be a manifestation of early autonomic failure. Further discussion of this group is outside the scope of this paper.

Upon standing from a supine or seated position, healthy, normally volume-replete individuals will typically experience a modest decline in systolic blood pressure (<10 mmHg), a very slight increase in diastolic blood pressure (approximately 2.5 mmHg) and a modest increase in heart rate (10–20 beats per minute [bpm]) [6]. These compensatory changes are induced primarily by augmentation of norepinephrine output by sympathetic autonomic peripheral nerve endings. Measuring the heart rate change from the supine (and/or seated) to standing may aid in differentiating between nOH and OH [35]. If an individual develops OH upon standing, an increase in heart rate of <15 bpm suggests a diagnosis of nOH. In contrast, individuals with non-neurogenic OH will typically demonstrate an increase in heart rate of >15 bpm within 3 min of standing [7, 36]. The heart rate information is acquired during the measurement of orthostatic vital signs as described above. It should be noted that without a fall in blood pressure, the heart rate criteria for nOH do not apply. In addition, monitoring of postural heart rate changes for diagnostic purposes requires that there is no confounding medication effect (e.g., beta blockers, alpha–beta blockers, non-dihydropyridine calcium channel blockers; see below) or intrinsic cardiac rhythm disturbances (e.g., sick sinus syndrome, complete heart block, dependence on a mechanical pacemaker) that prevent a compensatory heart rate increase upon standing. In a similar fashion, volume depletion (e.g., diuretics) in healthy individuals can be associated with OH, but should not be confused with nOH because there will still be an exaggerated increase in heart rate upon standing (≥20 bpm) in OH, contrasted with an expected failure to increase heart rate in nOH.

##### At-home monitoring of blood pressure and heart rate

In addition to in-clinic blood pressure monitoring, patients should be asked to check their blood pressure and heart rate at home and record the values in a diary. We recommend that patients or their caregivers check blood pressures at home in the supine position (at least 15 min after laying down at bedtime or prior to arising from bed in the morning) and after 3 min of standing (with assistance as necessary) after arising from bed in the morning. The following timings are our recommendations for at-home blood pressures and heart rate monitoring: (1) first thing in the morning before taking morning medications, (2) when a patient feels symptomatic, and (3) at bedtime for several days. We recommend that patients keep a blood pressure and heart rate diary for at least 7 days preceding a clinic visit, but after having a clinic visit patients should not need to measure blood pressure and heart rate on a continuous daily basis unless a change in therapy occurs. With any change in therapy, an additional week of blood pressure monitoring is required to determine the effectiveness of the change. (An example patient diary for blood pressure and heart rate is provided in Appendix I.)

##### Medication review

There are numerous medications that can diminish the normal compensatory postural increase in heart rate, (e.g., beta blockers, non-dihydropyridine calcium channel blockers, combined beta-/alpha-blockers such as carvedilol, central alpha-2 agonists such as clonidine or guanfacine, and antiarrhythmic agents such as amiodarone).

##### Exclude other causes of OH/nOH

If blood pressure and heart rate changes indicate OH/nOH, a complete history and physical examination, as well as electrocardiogram and laboratory testing, should be focused on ruling out non-neurogenic causes of OH, including cardiogenic, vascular, or iatrogenic etiologies [6, 7]. Table 2 lists recommended tests to conduct when evaluating a patient for OH/nOH. Cardiac disorders (e.g., pacemakers, dysrhythmias, ablation, etc.) can preclude heart rate augmentation [37]; therefore, a cardiac history, an electrocardiogram, and medication review (as noted above) should consistently be performed. Due to variability among patients, including age, disease status, and current medication usage, the lack of heart rate increase on standing may not always be an accurate indicator of nOH in patients with OH; therefore, clinical scenario and specific presentation need to be considered.

**Table 2:** Recommended initial testing to evaluate individuals presenting with OH/nOH

Test	Function in OH/nOH differential diagnosis
Electrocardiogram	To evaluate cardiac electrical activity
Complete blood count (CBC)	To evaluate for anemia, or infection that could contribute to non-neurogenic OH
Basic metabolic panel (sodium, potassium, chloride, bicarbonate, blood urea nitrogen, creatinine and fasting glucose)	To look for hypo/hypernatremia, hypo/hyperkalemia, acid–base disorders, blood volume depletion (BUN:Cr ratio >20 mg/dL:1 mg/dL), renal dysfunction or diabetes
TSH	To evaluate for thyroid dysfunction
B_12_ level, Methylmalonic acid	To look for evidence of B_12_ deficiency

Secondary Laboratory Tests (Considered for Use in Select Patients)	Function in OH/nOH differential diagnosis
Albumin	To identify poor nutrition or chronic illness
Liver enzyme testing, albumin	To evaluate for hepatic dysfunction in patients with weight loss and constitutional symptoms
Neurological antibody studies (paraneoplastic panel)	To identify autoantibodies; rarely indicated; only in patients with subacute onset of nOH in the presence of other neurological or constitutional symptoms suggesting an autoimmune or paraneoplastic syndrome. A pure autonomic failure syndrome should be tested for anti-ganglionic acetylcholine receptor antibodies
Serum and urine protein electrophoresis	To identify a monoclonal gammopathy; only in patients with features of peripheral neuropath

##### Specialty testing

If standard orthostatic blood pressure testing does not reveal OH in an at-risk individual with unexplained postural symptoms, falls, or syncope, then any of the following are appropriate as next steps: (1) conducting extended at-home blood pressure monitoring with results recorded by the patient or caregiver, (2) implementing 24-h ambulatory blood pressure monitoring where the patient can annotate times when supine or standing (to determine a typical range of blood pressures during the day and, with patient annotation of position, help to understand fluctuations), or (3) use of autonomic function tests. Some individuals may develop delayed OH later than 3 min of standing, which can be identified with prolonged standing blood pressures, or prolonged tilt table testing (HUT) [34, 38].

The specialized tests described below are considered by the panel to be beyond the scope of many primary care clinicians and usually require referral to a center specializing in autonomic disorders. Specialized tests for a definitive diagnosis of nOH include autonomic reflex testing (including heart rate variability to paced breathing, heart rate and blood pressure response to a Valsalva maneuver, and continuous blood pressure response to a prolonged HUT). Other tests used to help diagnose nOH may include plasma fractionated catecholamine levels and/or sudomotor function testing.

These specialized tests may be helpful in confirming the diagnosis of nOH by demonstrating baroreflex dysfunction based on HUT testing with beat-to-beat blood pressure measurement, and/or the beat-to-beat blood pressure and heart rate response to the Valsalva maneuver [39]. In addition, cardiovascular reflex testing, tilt table testing, and the Valsalva maneuver may help discriminate multiple system atrophy with predominant parkinsonism (MSA-P) from PD [40]. Supine and standing plasma fractionated catecholamine levels can aid in making a diagnosis, but have somewhat low sensitivity [6, 41]. At-home, 24-h blood pressure monitoring may be helpful not only to discern hypotensive episodes throughout the day (as long as patients are strongly encouraged to keep a diary of posture/activity), but also to identify supine hypertension, which is commonly seen in patients suffering autonomic dysfunction, whether or not they are experiencing OH [7].

### If the diagnosis is OH and not nOH

Non-neurogenic causes for OH are common and frequently include effects of medication (antihypertensive agents, antidepressants, and alpha-blockers being the most common; a comprehensive list is noted in Table 3), blood volume depletion, or chronic illness with deconditioning. It is useful to differentiate between OH and nOH because of the significantly greater morbidity and mortality associated with nOH [3, 6]. Many of the diagnosis and management points noted throughout this paper are appropriate for use in all individuals with OH. Readers are referred to several comprehensive reviews for further information [22, 33, 42]. A full review of the diagnosis and management of non-neurogenic OH is outside the scope of this paper.

**Table 3:** Common medications that may cause OH or exacerbate the symptoms of nOH

Class of medications	Common examples
Dopaminergic agents	Levodopa, dopamine agonists
Antidepressants (particularly tricyclic agents)^a^	Amitriptyline, nortriptyline, imipramine, desipramine
Anticholinergics	Atropine, glycopyrrolate, hyoscyamine
Anti-hypertensive agents	
*Preload reducers*	
Diuretics^a^	Furosemide, torsemide, acetazolamide, hydrochlorothiazide, spironolactone
Nitrates^a^	Nitroprusside, isosorbide dinitrate, nitroglycerin
Phosphodiesterase E5 inhibitors	Sildenafil, vardenafil, tadalafil
*Vasodilators*	
Alpha-1 adrenergic antagonists^a^	Alfuzosin, doxazosin, prazosin, terazosin, tamsulosin (used primarily for benign prostatic hyperplasia)
Dihydropyridine calcium channel blockers	Amlodipine, nifedipine, nicardipine
Other direct vasodilators	Hydralazine, minoxidil
*Negative inotropic/chronotropic agents*	
Beta-adrenergic blockers	Propranolol, metoprolol, atenolol, bisoprolol, nebivolol (also vasodilator), carvedilol (also alpha-1 antagonist), labetalol (also alpha-1 antagonist)
Non-dihydropyridine calcium channel blockers	Verapamil, diltiazem
*Central sympatholytic agents*	
Centrally acting alpha-2 agonists	Clonidine
False neurotransmitters	Alpha-methyldopa
*Renin–angiotensin system (RAS) antagonists*	
Angiotensin converting enzyme (ACE) inhibitors	Captopril, enalapril, perindopril,
Angiotensin receptor type II blockers (ARB)	Losartan, telmisartan, candesartan

^a^Agents that may cause more significant worsening of OH/nOH

### Grading of nOH after diagnosis

After making a diagnosis of nOH, it is was the opinion of the panel that it is important to establish its severity. A proposed grading scale for nOH is based on the total drop in systolic blood pressure, duration of standing time, and the number and severity of symptoms that affect activities of daily living [29]. The proposed grading scale is presented in Table 4. The purpose of the grading scale is to help clinicians decide when to refer a patient to a specialist. For example, depending on comfort level, a patient presenting with Grade 1 or 2 may not require referral; whereas, consideration of referral for a patient presenting with Grade 3 or 4 nOH is reasonable.

**Table 4:** Proposed grading scale for nOH [27]

Grade	Attributes
1	Infrequent symptoms/unrestricted standing time AND mild OH [20-30 mmHg drop in SBP during supine-to-standing test]
2	≥5 min standing time (but not unrestricted) AND [> 30 mmHg drop in SBP OR moderate impact ADL]
3	<5 min standing time AND [> 30 mmHg drop in SBP OR severe impact on ADL]
4	<1 min standing time AND [> 30 mmHg drop in SBP OR incapacitated]

A patient with grade 3 or 4 nOH should be treated by a healthcare provider with experience in managing nOH

*SBP* systolic blood pressure, *ADL* activities of daily living

### Post-prandial hypotension

It should be noted that large meals, particularly those high in carbohydrates or associated with alcohol, can magnify the drop in blood pressure. Elderly persons are more susceptible to these effects [8]. If symptoms are more prominent postprandially, then measurement of orthostatic blood pressures before and after meals should be considered.

## Treating nOH

**Fig. 2:**
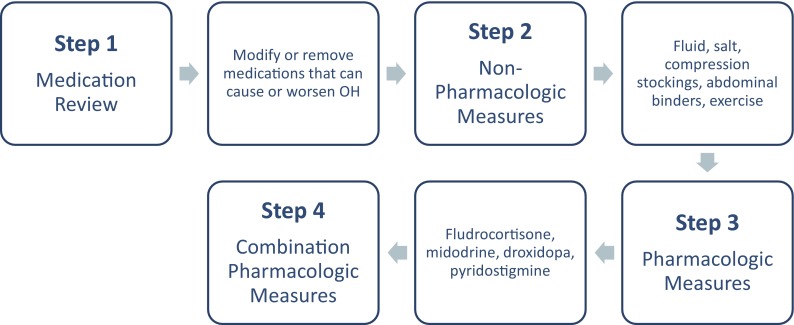
A 4-step process for treating nOH

Once a patient is diagnosed with nOH, the goal of treatment should not be to normalize standing blood pressure, but the principal treatment goals should serve to reduce the burden of symptoms (especially falls), prolong standing time, and improve the physical capabilities of the patient to restore independence in activities of daily living. A treatment algorithm for nOH that encompasses a 4-step hierarchical process is proposed (Fig. 2): (1) assessing and adjusting pre-existing medications, (2) utilizing non-pharmacologic approaches, (3) implementing single-agent pharmacologic treatment, and (4) with great caution, combining pharmacologic treatments. At each step, it is recommended that the patient undergo a 2-week assessment to establish whether sufficient symptomatic benefit has been achieved before moving onto successive steps. Each facet of the algorithm is described in detail below.

### Treating nOH—step 1: review and adjust current medications

After establishment of a diagnosis of symptomatic nOH, it is imperative to first consider pharmacologic simplification by reducing or discontinuing medication that exacerbate nOH. One of the keys to initial success is to complete a comprehensive medication review so that adjustments in regimens can be made as needed. Many medications (including those commonly used for treatment of PD, hypertension, or bladder symptoms) can lower blood pressure and exacerbate the symptoms of nOH (Table 3). Discontinuation or dose reduction of medications which can potentially aggravate orthostatic symptoms such as diuretics, vasodilators, and medications with negative chronotropic properties such as beta blockers may be sufficient to resolve symptoms of nOH in some patients.

Once a medication review has been conducted, it is recommended that any planned changes be discussed with the prescribing clinician such as: taking the patient off a particular drug, lowering current doses, or changing the dosing schedule. While there is limited published literature supporting this recommendation there is strong expert opinion underlying this approach. Following each adjustment to medication, changes to symptoms of nOH should be assessed, and this can be accomplished by asking the patient to respond to the screening questions listed in Table 1 [7, 43].

### Treating nOH—step 2: non-pharmacological measures

The next step in the treatment algorithm is to have the patient incorporate a number of simple non-pharmacological measures into their daily routines to address symptoms due to nOH. From a practical perspective, these measures are often incorporated into a treatment plan in parallel to the changes to pharmacology outlined in step 1 above. For patients who are experiencing syncope, near-syncope, or falls, there is some urgency to eliminating destabilizing postural changes. Hence, non-pharmacologic measures may be used individually, but are most effective when used in combination or while concomitantly titrating pharmacologic treatments.

#### Blood volume repletion

Patients with nOH require interventions which are aimed at ensuring normal or even expanded blood volume. Many patients with nOH, especially older patients, are often blood volume depleted due to inadequate oral fluid intake [44]. This may be due to voluntary restriction of intake for self-management of common conditions causing urinary urgency and urinary frequency including benign prostatic hyperplasia (BPH), overactive bladder, neurogenic bladder, stress incontinence or similar bladder dysfunctions as are commonly seen in many neurodegenerative disorders. However, the most common identifiable and readily treatable problem is decreased daily water intake. Most patients are unaware of the volume of water intake necessary during a typical day. A minimum of 64 oz (approximately 2 L) of water daily is recommended to achieve adequate daily hydration, although many clinicians recommend over 100 oz (3 L) daily to ensure blood volume repletion, depending on cardiac status. Modifications in fluid volume recommendations also need to be considered in geographic areas with warmer weather or during the summer season.

In addition to maintaining intravascular blood volume and to support standing blood pressure, patients with nOH who rapidly consume (within 5 min) 16 oz (approximately 500 ml) of free water can raise systolic blood pressure by 30 mmHg within 5 min [45]. The effect is due to a hypo-osmolar reflex in the portal circulation and can last for an hour to help alleviate the symptoms of nOH experienced on standing [46, 47]. Liquids other than water do not provide the same blood pressure response [48, 49]. Thus, proper hydration can produce both acute and long-lasting significant clinical benefits to patients with nOH [46, 50–53].

#### Salt intake

Another non-pharmacologic treatment is to monitor and adjust as needed the amount of salt that the patient is ingesting. Because salt is typically seen as a negative dietary component, patients may try to remove or at least reduce salt from their diet. However, many patients who suffer orthostatic symptoms have an inadequate intake of salt. This can be verified by checking the 24-h urinary sodium (in patients who are not taking diuretics or fludrocortisone, urinary sodium >100 mEq/24 h indicates salt repletion). For the patient with nOH, it is recommended that they add up to 1–2 teaspoons (2.3–4.6 g) of salt per day to their normal diet [54–56]. Patients at risk for heart failure or severe peripheral edema must be closely monitored for worsening symptoms and salt intake adjusted downward accordingly [43, 57]. The long-term risks associated with greater salt intake (e.g., increased intravascular volume, worsening edema, worsening heart failure, increased blood pressure) need to be weighed against the short-term risks of nOH resulting in fall injury and the negative impact on activities of daily living. The long-term risks of high sodium diets in individuals with orthostatic hypotension have not been well studied.

#### Physical conditioning

Lower body strength training and moderate, non-strenuous activities may be incorporated into standard treatment for patients with nOH [58, 59]. Deconditioning occurs very quickly in bed bound or hospitalized patients and will exacerbate the magnitude of the blood pressure drop in patients with nOH. We advocate the use of exercise that is not gravitationally challenging, such as a stationary recumbent bicycle, rowing machine, or water-based activities. Upright exercise, such as treadmill walking or running, should be avoided in some patients because of the risk of falls due to nOH. Patients should be cautioned that strenuous activity may temporarily exacerbate symptoms of nOH due to increased core body temperature and peripheral vasodilation as described below. To help mitigate against this occurring, patients should be well hydrated prior to exercise and should be careful when standing after an exercise session.

#### Avoid increased core body temperature

Elevation in body temperature causes peripheral vasodilation. Patients with nOH should avoid situations that could increase core body temperature, such as excessive high-intensity exercise, exercise when ambient temperature and humidity are high, utilization of hot tubs, spas, or saunas, prolonged hot showers, etc. [60]. Simple safety adjustments, such as using a shower chair, will help prevent complications. Additionally, individuals with autonomic failure may have impaired thermoregulatory capacity and may be at increased risk for hyperthermia. An example of impaired thermoregulation in autonomic failure is shown by the lack of the expected nocturnal decrease of body core temperature that has been described in patients with MSA [61].

#### Head-up position while sleeping

Elevating the head of the bed (through use of a wedge under the mattress, or placing blocks under the legs of the bed’s headboard so that the head is 6–9 inches (15–23 cm) higher than the feet—stacked pillows are not adequate) will reduce supine hypertension [62]. Supine hypertension commonly leads to a pressure diuresis that occurs resulting in nocturia and blood volume depletion overnight. This nocturnal forced diuresis can be decreased by elevating the head of the bed. In addition, the modest effects of gravity in the head-up position will maintain activation of the renin–angiotensin–aldosterone system and maintain higher blood pressure in the morning. The net result is a diminished magnitude of blood pressure drop in the morning [63].

#### Compression garments

Compression garments are another mechanism to combat blood pressure changes due to postural venous pooling [64, 65]. Compression of 30–40 mmHg is required to improve venous return and provide a meaningful blood pressure impact. Because most of the pooling occurs in the splanchnic–mesenteric bed, waist-high compression garments are the most effective, followed by thigh-high compression stockings. Knee-high stockings are not effective, although they are widely used for treatment of orthostatic hypotension and many patients are convinced of their effectiveness. Unfortunately, compliance with the use of compression stockings is low because they require fitting, are difficult to put on, and uncomfortable in hot climates. Abdominal binders offer an effective alternative [66], and arguably should be tried first, alone or if necessary in combination with leg compression. It was recently shown that an automated inflatable abdominal binder that supplied 40 mmHg in compression was as effective as midodrine in managing OH in patients with autonomic failure [67].

#### Diet

In patients with OH/nOH, normal sympathetic activity cannot compensate for blood pooling within the splanchnic circulation after eating. With nOH, sympathetic vasoconstrictor nerve activity is deficient and many patients become severely hypotensive within 2 h of eating [8, 68]. It is important to recognize this problem because treatment of OH/nOH can diminish symptoms post-meal. Patients can be asked to measure their blood pressure before and 30 min after a high carbohydrate meal. In individuals with postprandial hypotension, smaller, more frequent meals are recommended [69, 70]. There is also some evidence that a low glycemic diet may have a beneficial effect on the symptoms of OH/nOH [68, 71, 72]. Finally, postprandial hypotension can be reduced with caffeine [73] or acarbose [74].

#### Anemia and vitamin/mineral deficiencies in the diet

Anemia leads to decreased blood viscosity and oxygen carrying capacity and may worsen symptoms of OH/nOH [75]. Vitamin B12 deficiency (<250 pg/mL with elevated methylmalonic acid levels) may be also be associated with postural instability and can cause OH [76, 77]. B12 deficiency and anemia should be corrected and ongoing observation is necessary to prevent recurrence. Thus, changes in diet as well as vitamin and iron supplementation may be helpful for some patients with nOH.

### Treating nOH—step 3: initial pharmacologic treatment

If the implementation of non-pharmacologic measures does not adequately improve the symptoms of nOH, then it becomes necessary to initiate pharmacotherapy. For patients who are experiencing syncope, near-syncope, or falls, the potential consequences are so grave that some clinicians believe that institution of pharmacotherapy at the outset of management is appropriate. Clinicians must individualize treatment based upon the urgency of the symptoms.

Until 2014, the two drugs primarily used to treat nOH were fludrocortisone and midodrine (of which only midodrine has received FDA approval for treatment of OH). In 2014, droxidopa received FDA approval for the treatment of nOH. The selection of one drug over the other, in many situations, was related to clinician preference and experience. Below, we present an overview of the key drugs used to treat nOH and the recommendations for usage. One of the challenges associated with treating nOH pharmacologically is the limited availability of clinical evidence and lack of comparative effectiveness studies.

### FDA-approved drugs for the treatment of OH/nOH

#### Midodrine

Midodrine is a prodrug whose metabolite, desglymidodrine, is an α 1-adrenoreceptor agonist that increases vascular resistance and blood pressure. Typical dosing is between 2.5 and 15 mg once to three times daily during waking hours (an example schedule of three times daily would be dosing prior to getting out of bed, before lunch, and mid-afternoon) [7]. The dose is typically up-titrated to symptomatic relief. In multiple clinical trials, midodrine resulted in a significant increase in systolic and diastolic blood pressure, as well as modest improvements in orthostatic symptoms [11–13]. Midodrine carries a risk of significant supine hypertension; so it is recommended that individuals not take midodrine within 5 h of bedtime [11–13]. A high supine blood pressure seen shortly after midodrine administration should not cause the drug dose to be decreased or stopped, but managed by avoiding the supine posture. A meta-analysis of seven trials with midodrine (325 total subjects) found an increased incidence of supine hypertension and that the pooled risk ratio was 6.38; however, it is worth re-stressing that patients treated with midodrine should not rest or sleep in the supine position; rather, recumbency should always be assumed in the head-up position [78]. Other side effects with midodrine include piloerection, scalp itching, and urinary retention [79]. Caution should also be exercised in patients with congestive heart failure and chronic renal failure [79].

#### Droxidopa

Droxidopa is an orally administered norepinephrine pro-drug that is converted into norepinephrine both in the central nervous system and in peripheral tissues, including sympathetic peripheral nerve endings. Increases in circulating plasma norepinephrine levels peak at 6 h following treatment with droxidopa and levels of norepinephrine remain elevated for at least 46 h [80]. Replenishment of neural norepinephrine is believed to be the primary mechanism of action for improvement of standing blood pressure with droxidopa. Droxidopa can be dosed from 100 to 600 mg three times daily during waking hours. A recommended dosing schedule would be at 8 AM, noon, and 4 PM. In the clinical trials, droxidopa was titrated every 48 h to either symptomatic benefit and/or intolerable adverse events. Clinically, droxidopa has been evaluated in Phase 3 studies and has demonstrated significant improvement in the symptoms of nOH such as dizziness, lightheadedness, weakness, fatigue, and in improvements to activities of daily living [15–17]. Additionally, in studies of droxidopa for the treatment of nOH, the rate of falls and fall-related adverse events demonstrated a favorable trend (but not statistically significant) in the groups of patients receiving droxidopa versus those receiving placebo [15–17]. A post hoc analysis of the most recent study of droxidopa for the treatment of nOH showed that the group treated with droxidopa had 68% fewer falls than the placebo group (229 vs. 716) [17]. Droxidopa, like other agents to treat nOH, is not recommended to be taken within 5 h of bedtime to avoid the risk of supine hypertension. Side effects observed with droxidopa included headache, dizziness, nausea, fatigue, and supine hypertension [15–17]. Caution should be exercised in patients with congestive heart failure and chronic renal failure.

### Off-label use of FDA-approved drugs for the treatment of orthostatic hypotension

#### Fludrocortisone

Fludrocortisone has been used off-label for many years for the treatment of OH/nOH, and even though the level of data supporting the use of fludrocortisone in OH/nOH is low, it is included in treatment guidelines based on expert opinion [22]. It acts by increasing renal sodium and water reabsorption, thus expanding intravascular blood volume. Fludrocortisone’s long-term efficacy, however, may be related to increased vascular resistance. Fludrocortisone is typically dosed at 0.1–0.2 mg/day with little benefit being observed with increasing the dose beyond 0.3 mg/day (and an associated increase in risks of side effects, including hypothalamic–pituitary–adrenal axis suppression) [81–84]. The onset of action occurs over 3–7 days. The main side effects of fludrocortisone include supine hypertension, hypokalemia, and edema. It should be used with caution in patients with congestive heart failure [22].

#### Pyridostigmine

Pyridostigmine is an acetylcholinesterase inhibitor that has been used off-label for the treatment of OH/nOH because it potentiates neurotransmission at peripheral cholinergic synapses including those in the sympathetic ganglia. Pyridostigmine is thought to work in OH by amplifying the increased sympathetic nerve activity in response to orthostatic stress. Therefore, it is likely more useful in less severe patients with residual sympathetic function, and has the advantage of not worsening supine hypertension. Typical dosing is 30–60 mg once to three times per day. Several small studies have reported a modest improvement in OH and orthostatic symptoms [14, 18–21]. Patients may experience adverse side effects associated with cholinergic stimulation, including abdominal cramps, diarrhea, sialorrhea, excessive sweating, and urinary incontinence. Many patients with nOH also have autonomic failure resulting in constipation and anhidrosis, so these side effects may be salutary for some patients.

### Recommendations for initiating nOH treatment

There have been no head-to-head comparison studies to guide the initial choice of nOH treatments. An individualized treatment regimen should consider severity, co-morbid disease (especially cardiac or renal failure), and treatment goals. Midodrine has an FDA-approved indication for the treatment of symptomatic OH; FDA approval was based on studies showing an improvement in upright blood pressure as a surrogate for symptom relief. In contrast, the FDA-approved droxidopa with an orphan designation for the treatment of nOH based on studies showing improvement in symptoms of nOH. Patients receiving droxidopa reported a decrease in dizziness, lightheadedness, feeling faint, or feeling as if they might black out compared with those receiving placebo. There have been no long-term studies on the durability of the treatment effect for either midodrine or droxidopa. However, a long-term study is underway to study durability of effectiveness with droxidopa (NCT02586623) in patients with nOH for up to 36 weeks of treatment.

### Recommendations for changing nOH treatment

Once initial therapy has begun, symptomatic benefit, including impact on activities of daily living, and changes in blood pressure need to be assessed frequently. If symptoms do not improve after reaching maximum labeled dose, it is recommended that the treatment be changed and symptomatic benefit be assessed once more. This process is iterative until either symptomatic benefit is achieved or maximum tolerable dose of the therapy is reached.

### Treating nOH—step 4: combination pharmacotherapy

Little data exists to determine efficacy and safety of different combinations of therapy compared to monotherapy for nOH. Based on the experience of the consensus panel, the recommendation is to appropriately titrate to maximum tolerable dose of a single agent and then, if symptomatic benefit is not obtained, consider switching to a different therapy or adding a second agent and titrate from its lowest starting dose.

### Assessing nOH treatment success

Assessment of treatment success needs to be a multifaceted approach involving predominantly the measurement of symptomatic improvements as well as ongoing blood pressure measurements. It is critical that patients are educated on the various symptoms and about how to keep a diary of symptoms. Similarly, it is necessary for the patient to conduct a period of home monitoring of blood pressure after implementing new or additional non-pharmacologic measures or after a change in treatment or dose. We recommend that patients check their blood pressures at home in the supine head-up position (at bedtime, prior to arising in the morning, and while the patient is in their normal head-up sleeping position). Patients taking pressor medications such as midodrine or droxidopa should avoid the supine position during the 4–5 h after taking medication), and check blood pressure after 3 min of standing. We recommend checking blood pressures first thing in the morning prior to taking any morning medications, when symptomatic, and at bedtime for several days. The blood pressure diary should be evaluated by the healthcare provider 2 weeks after any therapeutic change to determine the need for further adjustments. (An example patient diary for blood pressure and heart rate monitoring is provided in Appendix I). If blood pressure measurements have been stable, a reduction in monitoring frequency can be considered, but reinstituted if symptoms worsen or if medications are changed. Lastly, 24-h ambulatory blood pressure monitoring can be considered so that there is an ongoing record of the impact of treatment on blood pressure. Additional clinical assessments will be largely driven by symptom frequency and severity. However, re-assessment of the clinical condition should occur at every visit via symptom review and orthostatic blood pressure measurements.

### Referring the nOH patient

With appropriate education, the screening, diagnosis, and treatment of nOH are well within the purview of both primary and specialist clinicians. With a diagnosis of nOH, the clinician can assign an OH severity Grade based on blood pressure changes, symptoms, and impact on activities of daily living. Once diagnosis is established, the clinician can take the patient through steps 1, 2, and 3 of the treatment algorithm. However, if the patient experiences pharmacotherapy failure, it may be useful to refer the patient to a specialist who is experienced in treating patients with nOH. Additionally, if the clinician is at any time uncomfortable with treating a patient with nOH of high severity (Grades 3 to 4), they should refer the patient to a specialist with expertise in the treatment of nOH.

## Supine hypertension

### Defining supine hypertension in patients with nOH

According to the Eighth Joint National Committee (JNC8) hypertension guidelines, essential hypertension is a blood pressure consistently ≥140/90 mmHg [85]. Supine hypertension in nOH patients is arbitrarily defined as a systolic blood pressure ≥150 mmHg or diastolic blood pressure ≥90 mmHg while in the supine position. General treatment guidelines recommend intervention for hypertension, but supine hypertension associated with nOH requires additional considerations. In patients with autonomic failure causing nOH, supine hypertension is common and part of the underlying disease process since these patients lack the normal blood pressure buffering mechanisms that offset hypertension. In addition, frequent periods of OH may lead to chronic activation of the renin–angiotensin system.

A supine systolic blood pressure of up to 160 mmHg should be monitored but does not generally warrant treatment, especially if the symptoms of nOH have improved. The long-term risks associated with supine hypertension need to be balanced with the short-term risks of OH. Expert recommendations for the management of supine hypertension in the setting of nOH suggest that supine hypertension requires intervention if systolic blood pressure exceeds the range of 160–180 mmHg. However, it should be noted that individuals with the largest drops in blood pressure upon standing (>80 mmHg drop) will require significantly higher supine blood pressures in order to achieve a standing position, and therefore permissive supine hypertension may need to be tolerated.

### Measuring supine hypertension in patients with nOH

The first step in evaluating supine hypertension in the patient with nOH is obtaining a series of blood pressure measurements. Home supine and standing blood pressures should be obtained. The supine assessment should be in the morning (before arising) and at bedtime with the patient in their normal sleeping position and with the head of the bed raised. If necessary, 24-h blood pressure monitoring may be warranted in some patients. Blood pressure should be monitored for a week or more to establish a pattern of typical blood pressure. Once a baseline has been established and treatment initiated for nOH, regular blood pressure monitoring for a period of 2 weeks following treatment initiation is crucial to gauge the impact of treatment on supine hypertension.

### Supine hypertension associated with nOH

It is critical for clinicians to understand the physiology underlying both nOH and supine hypertension and the associated risks of each when they manage patients with nOH. Neurogenic OH is associated with loss of baroreflex function that normally buffers changes in blood pressure in both directions. Therefore, patients with autonomic nervous system dysfunction typically have nOH and supine hypertension. In addition to supine hypertension being common in patients with nOH, many of the medications used to treat nOH can cause or exacerbate supine hypertension. Because OH/nOH and supine hypertension are hemodynamic opposites, improving one can worsen the other. Thus, all patients with nOH should be evaluated for supine hypertension. There are differences in clinical practice among clinicians who treat nOH. Some clinicians may not treat nOH for fear of exacerbating supine hypertension, while others may aggressively treat nOH while accepting the resulting supine hypertension. Unlike the potential long-term consequences of hypertension in non-PD patients, the risks of nOH are immediate and represent potential major health threats. By extrapolation, similar recommendations can be made in multiple system atrophy [3, 4]. In contrast, the data is less clear in individuals with pure autonomic failure.

In most patients with nOH, there are strong reasons for prioritizing the treatment of nOH over supine hypertension [10, 86]. Symptomatic nOH carries a variety of debilitating symptoms including postural related dizziness, syncope, fatigue, weakness, and vision impairment. All of these symptoms can contribute to an increase in the occurrence of falls, which is one of the most common reasons for hospital admission for PD patients. Falls due to nOH can lead to numerous complications that can result in death. However, there is no agreement among clinicians, when, or how vigorously supine hypertension should be treated with nOH and there is no clinical study evidence to base guidelines. At the very minimum, all patients with nOH and supine hypertension should be advised to avoid supine posture during the day and elevate the head of the bed as tolerated during the night. Clinicians should manage patients with significant supine hypertension with short-acting antihypertensive agents given at bedtime and avoid fludrocortisone. Since patients with MSA have a short life expectancy and experience rapid deterioration of motor abilities, enhancement of quality of life should take precedent and nOH should be aggressively treated to allow for improved mobility in these patients. However, once an MSA patient becomes wheelchair bound there may be less urgency to treating nOH. In patients with PD and PAF the decision to prioritize the treatment of nOH over supine hypertension should be individualized. If supine hypertension can be treated without worsening nOH, the clinician should consider using short-acting agents at night. Lastly, those few patients who have episodes of seated hypertension during the day should be referred to specialized centers.

As stated above, if patients with nOH are experiencing supine hypertension (systolic blood pressure of 160–180 mmHg or diastolic blood pressure of 90–100 mmHg), the decision to treat should be individualized. However, if patients with nOH are experiencing severe supine hypertension (systolic blood pressure of >180 mmHg or diastolic blood pressure of >110 mmHg), they could be treated in the evening using short-acting anti-hypertensive agents (Table 5). Individuals with the most severe supine hypertension often have the most severe nOH and therefore these patients should be educated in the risks of worsening nOH with treatment. In particular, nocturnal visits to the bathroom may become dangerous if supine hypertension is treated too aggressively. It is also important for these patients to avoid the use of diuretics and long-acting antihypertensive agents because the blood volume depletion attendant to diuretics and hypotensive effects of long-acting antihypertensive may markedly worsen nOH, even though they might have been intended to mitigate supine hypertension. Additionally, patients with nOH must avoid the supine posture during the day. A head-up reclining posture is recommended for repose.

**Table 5:** Proposed treatments for supine hypertension related to nOH

Treatment options*	Mechanism of action	Typical dose
Captopril	ACE inhibitor	25 mg qhs
Clonidine^a^	Central *α*-2 agonist	0.2 mg with evening meal
Hydralazine	Peripheral smooth muscle relaxant	10–25 mg qhs
Losartan	Angiotensin II receptor antagonist	50 mg qhs
Nitroglycerine patch	Vasodilator	0.1 mg/h patch qhs (remove patch in AM)

*Short-acting antihypertensive medications for treatment of supine hypertension should only be administered at bedtime, not during daytime hours. Many medications have BID or TID as the recommended dosing regimen, and patients may inadvertently start taking these medications during daytime hours and worsen symptoms of nOH

^a^Use of clonidine carries a risk of a morning ‘hangover’ effect

### Limitations

There are a number of limitations to the current publication. The recommendations listed in this document are based on expert opinion, but do not contain sufficient evidence to support official guidelines. However, the recommendations were included with the goal of providing practical information to the many physicians without specific training or experience in autonomic disorders that are most likely to manage patients with orthostatic hypotension. Additional studies are necessary to determine the utility of these recommendations.

## Conclusions

To date, little published literature is available on a “standardized approach” to the screening, diagnosis, and treatment of patients with OH/nOH and associated supine hypertension. As such, the recommendations of this panel, which were based on the discussions and experiences of the panel along with evidence in the literature, should provide clinicians with a useful working approach to dealing with this complex condition. The panel recommended that additional study in screening, diagnosis, and treatment of patients with OH/nOH and associated supine hypertension is necessary. In particular, additional studies of the treatment of nOH should be conducted to refine the diagnosis and treatment algorithms and to provide definitive evidence of efficacy and safety of medications that are currently used, but not approved, for the treatment of patients with nOH.

## Electronic supplementary material

Below is the link to the electronic supplementary material.

Supplementary material 1 (PDF 478 kb)
