# Supplementary material for: The recommendations of a consensus panel for the screening, diagnosis, and treatment of neurogenic orthostatic hypotension and associated supine hypertension
Source: J Neurol. 2017 Jan 3;264(8):1567–82. doi: 10.1007/s00415-016-8375-x (PMC5533816; doi:10.1007/s00415-016-8375-x)
Supplement: Supplementary file 1 — Supplementary material 1 (PDF 478 kb) [file 415_2016_8375_MOESM1_ESM.pdf]

APPENDIX 1: Example of a patient blood pressure and heart rate diary/log.

Date	Time	Position (circle)	Blood Pressure	Heart Rate	Symptoms

The Recommendations of a Consensus Panel for the Screening, Diagnosis, and Treatment of Neurogenic Orthostatic Hypotension and Associated Supine Hypertension

Journal of Neurology

Christopher H. Gibbons, MD;[1*] Peter Schmidt, PhD;[2] Italo Biaggioni, MD;[3] Camille Frazier-Mills, MD, MHS;[4] Roy Freeman, MD;[5] Stuart Isaacson, MD, PA;[6] Beverly Karabin, RN, PhD, CNP;[7] Louis Kuritzky, MD;[8] Mark Lew, MD;[9] Phillip Low, MD;[10] Ali Mehdirad, MD;[11] Satish R Raj, MD MSCI;[12] Steven Vernino, MD, PhD;[13] Horacio Kaufmann, MD[14]

1. Harvard Medical School and Beth Israel Deaconess Medical Center, Boston, MA
2. National Parkinson Foundation, Miami, FL
3. Vanderbilt University Medical Center, Nashville, TN
4. Duke University Hospital and Central Carolina Hospital, Durham, NC
5. Harvard Medical School and Beth Israel Deaconess Medical Center, Boston, MA
6. Parkinson's Disease and Movement Disorders Center of Boca Raton, Boca Raton, FL
7. University of Toledo Medical Center, Toledo, OH
8. University of Florida College of Medicine, Gainesville, FL
9. Keck Hospital, University of Southern California, Los Angeles, CA
10. Mayo Clinic, Rochester, MN
11. Saint Louis University Hospital, St. Louis, MO
12. University of Calgary, Calgary, BC
13. University of Texas Southwestern Medical Center, Dallas, TX
14. New York University Langone Medical Center, New York, NY

*Corresponding author: Christopher H. Gibbons, MD
Email: cgibbons@bidmc.harvard.edu
Telephone: 617-632-8454
Fax: 617-632-0852
